# Ensemble Machine Learning Model for Real-Time Valproic Acid Prediction in Epilepsy Treatment

**DOI:** 10.1055/a-2593-3125

**Published:** 2025-06-02

**Authors:** Jiangchuan Xie, Pan Ma, Xinmei Pan, Liya Cao, Ruixiang Liu, Lirong Xiong, Hongqian Wang, Xin Zhang, Linli Xie, Yongchuan Chen

**Affiliations:** 1 Department of pharmacy, The First Affiliated Hospital of Army Medical University, Chongqing, China; 2 Medical Big Data and Artificial Intelligence Center, the First Affiliated Hospital of Army Medical University, Chongqing, China

**Keywords:** valproic acid, machine learning, precision medicine, prediction model, model explanation

## Abstract

**Aims:**

To develop an optimal model to predict valproic acid (VPA) concentrations by machine learning, ensuring that the VPA plasma concentration is in the effective treatment range, and thus effectively control the patient’s epilepsy.

**Methods:**

This single-center, retrospective study included patients diagnosed with epilepsy from January 2014 to January 2022. Patients receiving VPA and having undergone therapeutic drug monitoring were enrolled. Top three algorithms exhibiting superior model performance were selected to establish the ensemble prediction model, with Shapley Additive exPlanations (SHAP) employed for model interpretation. An independent dataset was collected as a clinical validation group to verify the prediction model performance.

**Results:**

The algorithms chosen for the ensemble model—Light Gradient Boosting, Categorical Boosting, and Gradient Boosted Regression Trees—demonstrated high *R* ^2^ (0.549, 0.515, and 0.503, respectively). Post-feature selection, the final model incorporated 20 variables, proving superior in predictive performance compared to models considering all 24 variables. The *R* ^*2*^ , mean absolute error, mean square error, absolute accuracy (±20 mg/L), and relative accuracy (±20%) of external validation were 0.621, 10.67, 221.50, 78.98%, and 66.48%, respectively. The importance and direction of each variable were visually represented using SHAP values, with VPA administration and liver function emerging as the most significant factors.

**Conclusion:**

The innovative application harnesses advanced multi-algorithm mining methodologies to forecast VPA concentrations in adult epileptic patients. Furthermore, it employs SHAP to elucidate the nuanced influence of each feature within the integrated prediction model, thereby providing a robust and plausible explanation for the determinants affecting VPA concentration predictions.

## Introduction

Epilepsy affects 50–70 million people worldwide 1 , with the annual incidence being 50 in every 100,000. In China, the prevalence of epilepsy is about 7 ‰, increasing at a certain rate every year 1 2 . Epilepsy is a chronic brain disease characterized by seizures, with the risk of death in patients with epilepsy being two to three times higher than in the general population 3 . The International League Against Epilepsy defines “epileptic syndromes” as a group of specific disorders consisting of typical clinical and electroencephalographic features supported by a specific etiology 4 . Clinically, antiepileptic drugs are commonly used to control seizures.

Valproic acid (VPA), as a commonly used broad-spectrum antiepileptic drug, has the advantages of excellent efficacy and can be used as a first-line monotherapy for generalized seizures of all types of epilepsy and also as a combination therapy drug 5 6 . Experts suggest VPA as the first-line drug for three seizure types—generalized tonic-clonic seizures, apoplectic seizures, and myoclonic seizures—and its use of VPA has been widely promoted because of its clinical efficacy, rapid onset of action, and low recurrence rate 7 . The plasma concentration of VPA is closely associated with its therapeutic efficacy, and according to previous studies, 50–100 µg/ml was regarded as the target VPA level for successful treatment. However, a large number of studies have shown that various patients treated with conventional regimes may fail to reach therapeutic targets, leading to clinical failure 3 7 . VPA-albumin binding is commonly reported to be 90%; therefore, its concentration may be reduced in specific situations, such as the occurrence of hypoalbuminemia and azotemia, or the administration of medications that compete for binding, such as aspirin and phenytoin 8 . Additionally, the pharmacokinetic parameters of VPA are affected by age, hepatic and renal function status, drug combination, genetics, and other factors. Moreover, its therapeutic window is narrow and individual differences are obvious; thus, therapeutic drug monitoring (TDM) is recommended to ensure the efficacy and safety of VPA.

TDM is an effective method to monitor the concentration of VPA for maximum efficacy and thus prevent adverse effects resulting from overexposure. However, TDM is effective only after the drug reaches a steady state, making it difficult to obtain the concentration data in time for clinical practice. Due to limited hospital facilities and high experimental costs, more powerful drug concentration prediction tools are needed to support to clinicians and patients alike. Population pharmacokinetics (PPK) is the study of the sources of variation and correlation of drug concentrations between individuals and is a discipline that investigates the principles, computational methods, and applications of pharmacokinetic population parameters. Some studies have reported the use of TDM-assisted software such as JPKD and Smartdose to establish PPK models and predict drug plasma concentrations 9 10 . Although these software models provide valuable clinical insights, they often rely on static and linear assumptions, which may not fully capture the complexity of patient-specific dynamics. Consequently, the predictive accuracy of these traditional methods and applicability across diverse patient populations can be limited. In contrast, recent advancements in machine learning (ML) offer a more flexible and robust approach to drug concentration prediction. For instance, Soeorg et al. demonstrated that a Long Short-Term Memory neural network outperformed traditional pharmacometric models in estimating valproate concentrations using real-world data 11 . Likewise, Zhu et al. developed an interpretable stacking ensemble learning framework for real-time prediction of olanzapine concentrations, showcasing the potential of ML in personalized medicine 12 . These studies underscore the broader applicability and superior performance of AI-based tools in therapeutic drug monitoring, particularly in settings where traditional models fall short.

With the rapid advancements in artificial intelligence in the healthcare industry 13 , ML has become increasingly pivotal in precision medication 14 . Accurate prediction of drug concentrations facilitates optimal individualized treatment plans for patients. Hao employed the CatBoost algorithm to forecast the blood concentration of quetiapine in patients suffering from schizophrenia and depression 15 . Notably, the CatBoost model demonstrated marginally superior accuracy, achieving a prediction within±100% of the actual value, compared to the PBPK model. The researcher Ma P, et al. established a prediction model through a combination of ML and population pharmacokinetics for predicting teicoplanin concentrations, offering a non-invasive, rapid, and cost-effective method to estimate plasma levels in critically ill patients 16 .

The application of multi-algorithm mining techniques to predict VPA concentrations in epileptic adults has not been reported to date. ​In this study, we compared the performance of 12 ML algorithms, then developed an optimal ensemble model, and designed an easy-to-use, web-based application as a real-time assisted clinical decision support tool for personalized adjustment. In addition to developing an optimal model to predict VPA concentrations for effective epilepsy control, our secondary objectives include validating the model using clinical data to ensure its performance in real-world settings and exploring the potential for its real-time application as a clinical decision support tool. In this study, we used Shapley Additive exPlanations (SHAP) to elucidate the importance and influence of each feature in the ensemble prediction model for VPA plasma concentration. SHAP values quantify the contribution of each feature to the prediction outcome, with positive values indicating an increase in predicted VPA concentration and negative values indicating a decrease. This approach provides a clear and plausible explanation for the impact of relevant factors on the prediction of VPA concentration.

## Methods

### Patients and data

The trial was a single-center, retrospective study of both outpatients and inpatients diagnosed with epilepsy from January 2014 to January 2022 at the First Affiliated Hospital of the Army Medical University. Patients who received VPA and underwent TDM with valproate were enrolled. The following exclusion criteria were applied: (1) age<18 years, (2) pregnant women, and (3) irregular administration of VPA. Demographic data and laboratory data were recorded and collected.

### Ethics approval

This study was approved by the Hospital Ethics Committee of the First Affiliated Hospital of Army Medical University ([B]KY2023079) and performed in accordance with the Declaration of Helsinki. Informed consent has been waived in the ethical approval documents. The procedures in this study were fully compliant with the ethical standards of the Institutional Research Committee.

### Measurement of valproic acid trough concentration

Enzyme immunoassay was performed to measure the plasma trough concentration of VPA. All patients with epilepsy who have reached a steady-state concentration of the medication should have 2–3 mL of venous blood collected within 30 minutes prior to the next morning dose. Reagents used include the valproic acid (VPA) assay kit (Siemens AG), and the instrument used is the Siemens fully automated biochemical analyzer. The enzyme-amplified immunoassay method is employed to determine the concentration of VPA in the sample, through competition between VPA in the sample and the glucose-6-phosphate dehydrogenase (G6PDH) labeled VPA antibody in the reagents for binding sites on the antibody. Upon binding to the antibody, the activity of G6PDH decreased, and the concentration of VPA in the sample was measured based on the enzyme activity. This method quantifies VPA content in human serum or plasma up to 150 μg/mL, with a detection sensitivity below 1 μg/mL. The blood sample was collected after reaching steady-state concentration (continuous VPA administration for at least 3 days). The enzyme-multiplied immunoassay technique (EMIT) method was used to quantify VPA plasma concentration. The Syva Viva-ProE ^TM^ system (Siemens, Munich, Germany) combined with the VPA assay kit (4G019UL, Siemens) was used to determine the plasma concentration of VPA, which is a routine on the TDM platform of our hospital.

### Data collection and processing

Based on prior knowledge, the VPA dataset, including VPA administration, demographic information, laboratory parameters, concomitant therapy, and concomitant diseases, was obtained from the hospital’s Electronic Medical Record System. After cleaning up of the VPA dataset, the target variable and relevant crucial covariates were screened subsequently. The mean filling method in Python (version 3.8, Python Software Foundation) was employed to fill the missing values, resulting in a dataset of size 1222×25. The final outcome suggested VPA plasma concentration as the target variable, while the entire dataset was randomly divided into training and testing groups in a ratio of 8:2.

### Modeling and validation

Twelve common ML algorithms were used for modeling, including support vector regression (SVR), random forest (RF), Adaptive Boosting (AdaBoost), Bootstrap aggregating (Bagging), Gradient Boosted Regression Trees (GBRT) and eXtreme Gradient BoostingX (XGBoost), light gradient boosting machine (LightGBM), categorical boosting (CatBoost), back propagation neural network (BPNN), k-Nearest neighbors (KNN), Least Absolute Shrinkage and Selection Operator (LASSO), and ridge regression (Ridge). The optimal hyperparameters were searched by the grid.

To evaluate the predictive performance of the model, the metrics of R-squared ( *R* ^*2*^ ), mean square error (MSE), and mean absolute error (MAE) were used. *R* ^*2*^ indicates the explanation of the degree of the independent variable to the dependent variable. The calculation formulas were presented in our previous study 17 .

The predictive performance of a single algorithm was evaluated by fivefold cross-validation, and the top three algorithms were selected for the ensemble model. According to the importance ranking obtained by SHAP, the screening process selected the features that yielded the highest *R* ^*2*^ value for each algorithm, which were then included as the final features for each respective algorithm. As a result of feature selection, 20 variables were included in the ensemble model. Computer grid search was employed on the weight proportion (accurate to one decimal place) of the three algorithms, and the *R* ^*2*^ of each model was calculated.

**Fig. 1:**
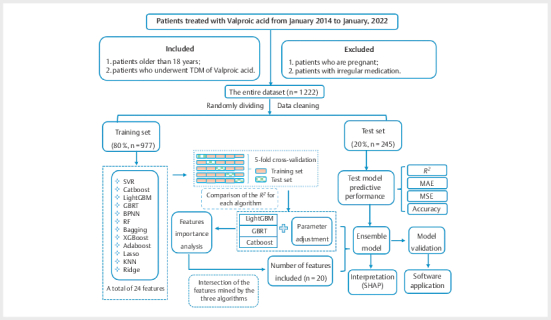
The workflow of data processing and algorithm selection. Abbreviations: LightGBM, light gradient boosting machine; CatBoost, categorical boosting; GBRT, gradient boosted regression trees; RF, random forest; Bagging, Bootstrap aggregating; XGBoost, extreme gradient boosting; Adaboost, adaptive boosting; SVR, support vector regression; LASSO: Least absolute shrinkage and selection operator; Ridge, ridge regression; KNN, k-Nearest Neighbors; BP, back propagation neural network.

To further evaluate the performance of the ensemble model, the accuracy of the predicted concentration was compared with the observed concentration. The absolute accuracy was defined as the accuracy of the predicted trough concentration to be within±20 mg/L of the observed trough concentration, while the relative accuracy showed that the predicted trough concentration was within±20% of the observed trough concentration. In addition, another dataset of 147 patients with 176 TDM measurements of VPA was collected as a clinical validation group to corroborate the performance of the prediction model. The workflow of data processing, algorithm selection, and modeling is shown in Fig. 1 .

### Model interpretation and software application

SHAP values were employed to interpret our ensemble prediction model. The SHAP Python package (version 0.39.0) and its permutation explainer were used to demonstrate the SHAP summary plot, importance ranking, SHAP dependence plot, and SHAP decision plot for the relevant covariates.

VPApredictor1.0, a software application based on the ensemble model, was developed with a user-friendly interface. As a calculator, VPApredictor1.0 allows users to input relevant clinical indicators and instantly obtain predicted plasma concentrations of VPA. Additionally, the SHAP force plot was incorporated into the output item, providing detailed analysis for individual predictions.

### Statistical analysis

Statistical analysis was performed using IBM SPSS version 25.0 (IBM Corp., Armonk, NY, USA). The Kolmogorov-Smirnov test was used to evaluate whether the measurement data were normally distributed. Measurement data are presented as the median and interquartile range for nonnormally distributed variables and the mean±standard deviation for regularly distributed variables and were analyzed by the Mann-Whitney U test (non-normal distribution) and independent t-test (normal distribution). Categorical data were expressed as n (%) and analyzed using the Chi-square test. The tests were two-sided, with a *p* -value of<0.05 deemed statistically significant.

## Results

### Baseline patient characteristics

Patients treated with VPA from January 2014 to January 2022 were included in this study. A total of 1,222 data were collected from 749 patients and randomly divided into two groups, a training group and a test group, with a ratio of 8:2, respectively. Patients who were 18 years old and above, and those who underwent TDM of VPA, were included, while pregnant women and patients with irregular medication were considered ineligible. The study included two dosage forms of VPA, both oral and injectable, with more than 80% of patients taking daily doses greater than or equal to 1 g. The dataset was collected from patients in the Department of Neurology, Epilepsy Department, Intensive Care Unit, and Rehabilitation Physiotherapy Unit. The baseline information of 24 variables and the comparison between the training and testing groups, in particular laboratory parameters, has little to no significant difference (p>0.05), as shown in Table 1 .

**Table:** **Table 1** The description of the study samples.

Variables	Values		P-value
	Training (n=977)	Testing (n=245)	
Valproic acid concentration (mg/L)	68.92±22.02	67.51±23.03	0.861 ^b^
Valproic acid administration			
Total daily dose (g)			0.698 ^c^
< 1 g	177 (18.12)	39 (15.92)	
1 g	377 (38.59)	95 (37.78)	
> 1 g	423 (43.29)	111 (45.31)	
Way of administration			0.452 ^c^
Oral administration	592 (60.59)	142 (57.96)	
Injection administration	385 (39.41)	103 (42.04)	
Demographic information			
Age (years)	45 (32, 55)	46 (31.5, 56)	0.728 ^a^
Gender			0.836 ^c^
male (n,%)	633 (64.79)	157 (64.08)	
Female (n,%)	344 (35.21)	88 (35.92)	
Weight	64.41 (55.82, 64.41)	64.41 (55.88, 64.41)	0.790 ^a^
Height	167.43 (156.32, 167.43)	167.43 (156.32, 167.43)	0.873 ^a^
Laboratory parameters			
GGT (IU/L)	29 (18, 53)	29 (18.55, 59.4)	0.705 ^a^
ALT (IU/L)	19.7 (12.15, 31.75)	21 (13, 33)	0.184 ^a^
AST (IU/L)	24.2 (18.05, 34.05)	24.2 (19.05, 41.1)	0.301 ^a^
ALP (IU/L)	78 (62, 96)	78 (65, 108)	0.080 ^a^
TBIL (umol/L)	10.5 (8.2, 14.3)	9.8 (7.6, 12.8)	0.003 ^a^
TBA (umol/L)	2.4 (1.5, 4.4)	2.4 (1.5, 4.1)	0.455 ^a^
TP (g/L)	66.7 (61.05, 71.9)	66.7 (61.6, 72.25)	0.500 ^a^
ALB (g/L)	38,1 (33.5, 42.9)	38.4 (34.15, 42.6)	0.458 ^a^
ALB/GLO	1.39 (1.16, 1.59)	1.39 (1.21, 1.59)	0.527 ^a^
UA (umol/L)	279 (196, 345.5)	279 (212.5, 351)	0.534 ^a^
Urea (mmol/L)	5.1 (4,6.4)	5.1 (4.14, 6.7)	0.329 ^a^
Cr (umol/L)	63.7 (52, 75.55)	63.7 (53.15, 78)	0.307 ^a^
eGFR (mL/min/1.73 m ^2^ )	111.52 (102.42, 120.54)	111.52 (96.78, 117.66)	0.154 ^a^
WBC (10 ^9^ /L)	7.50 (5.91, 9.52)	7.50 (6.03, 10.07)	0.506 ^a^
NEU%	65.7 (55.15, 77.75)	65.7 (52.75, 76.05)	0.409 ^a^
PLT (10 ^9^ /L)	188 (147.5, 232)	188 (152.5, 242.5)	0.362 ^a^
Combined with Carbapenem (n,%)			0.508 ^c^
Yes	76 (7.78)	16 (6.53)	
No	901 (92.22)	229 (93.47)	

Abbreviations: GGT, γ-glutamyltransferase; ALT, alanine aminotransferase; AST, aspartate aminotransferase; ALP, alkaline phosphatase; TBIL, total bilirubin; TBA, total bile acid; TP, total protein; ALB, albumin; ALB/GLO, albumin/globulin; UA, uric acid; Cr, creatinine; eGFR, estimated glomerular filtration rate; WBC, white blood cell count; NEU%, the percentage of neutrophils; PLT, platelet. Measurement data were presented as a median and interquartile range (IQR) for non-normal distribution variables and mean±standard deviation for normal distribution variables. Categorical data were expressed as n (%). ^a^ Mann-Whitney U test. ^b^ Independent t-test. ^c^ Chi-square test.

### Algorithm selection

The study incorporated 12 common ML algorithms. The *R* ^*2*^ values of different algorithms calculated by fivefold cross-validation are shown in Table 2 . Among the 12 algorithms, LightGBM, CatBoost, and GBRT had high goodness of fit, and their respective *R* ^*2*^ values were 0.549, 0.515, and 0.503. Therefore, the three best-performing algorithms were selected for subsequent experiments in this study to predict the replacement VPA concentration.

**Table:** **Table 2** The fivefold cross-validation of different algorithms.

Model	LightGBM	CatBoost	GBRT	RF	Bagging	XGBoost
***R*** ^***^2^***^	0.549	0.515	0.503	0.487	0.484	0.481
**Model**	AdaBoost	SVR	LASSO	Ridge	KNN	BPNN
***R*** ^***^2^***^	0.415	0.295	0.249	0.248	0.213	0.183

Abbreviations: LightGBM, light gradient boosting machine; CatBoost, categorical boosting; GBRT, gradient boosted regression trees; RF, random forest; Bagging, Bootstrap aggregating; XGBoost, extreme gradient boosting; AdaBoost, adaptive boosting; SVR, support vector regression; LASSO: Least absolute shrinkage and selection operator; Ridge, ridge regression; KNN, k-Nearest Neighbors; BP, back propagation neural network.

### Feature selection of variables

The top three ranking algorithms were visualized by SHAP and the important feature sets are shown in **Fig. S1** . Feature selection was determined by each algorithm itself and revealed a feature set in which the total daily dose was the most influential factor, followed by indices related to liver function and carbapenem, which is evident in the decrease in VPA plasma concentration. A forward stepwise inclusion method was employed for feature selection. Based on the SHAP importance ranking of each algorithm, features were gradually included in order of importance until the *R* ^*2*^ value reached a plateau. The feature combination at this point was considered an important feature set for the algorithm. As shown in **Fig. S1** , the highest *R* ^*2*^ value was achieved when LightGBM included 22 features, CatBoost included 21 features, and GBRT included 20 features. The intersection of these important feature sets (total daily dose, GGT [γ-glutamyltransferase], PLT [platelet], combined with carbapenem, ALT [alanine aminotransferase], gender, age, Cr [creatinine], ALB [albumin], urea, TBA [total bile acid], TBIL [total bilirubin], ALP [alkaline phosphatase], ALB/GLO [albumin/globulin], TP [total protein], WBC [white blood cell count], AST [aspartate aminotransferase], UA [uric acid], eGFR [estimated glomerular clearance], and NEU% [the percentage of neutrophils]) were included in the ensemble model as a result of feature selection. This approach ensured that only the most relevant and important variables were included in the final feature set.

### Modeling and validation

With 20 variables obtained by feature selection, the *R* ^*2*^ values of 66 permutations and combinations (from 0:0:1 to 1:0:0) of LightGBM, CatBoost, and GBRT were calculated, as mentioned in **Table S1** . This rigorous approach ensures that the ensemble model achieves the best possible performance by finding the optimal combination of weights for the used algorithms. Ultimately, the composition of LightGBM, GBRT, and CatBoost (5:3:2) was determined as the final ensemble model.

**Fig. 2:**
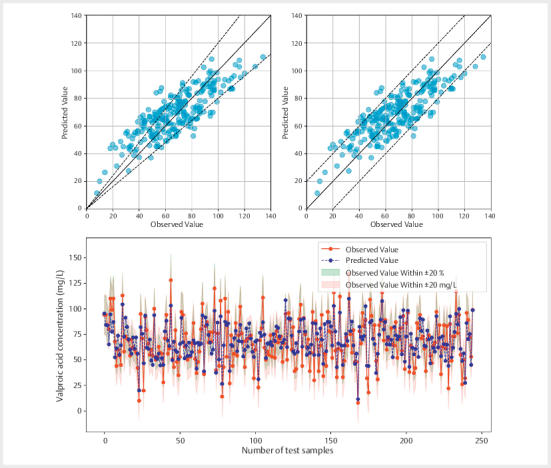
Comparison of predicted and observed values of the test group. The red dots indicated the observed values, and the blue dots indicated the predicted values. The pink shade represented within±20% of the observed values, and the purple shade represented within±20 mg/L of the observed values.

Ensemble models exploit the strengths of each algorithm and improve the overall prediction performance by combining their predictions. As shown in the data of the test group ( Table 3 ), the absolute accuracy (±20 mg/L) of the ensemble model was 88.16%, and the relative accuracy (±20%) was 64.49%, proving superior predictive performance even with 20 variables. The exact distribution of predicted and observed values for VPA plasma concentration in the testing group is shown in Fig. 2 .

**Table:** **Table 3** The comparison of model performance on test set and validation set in ensemble model.

Model	*R* ^*2*^	MAE	MSE	Accuracy-1 ^a^	Accuracy-2 ^b^
Test group ^#^	0.669	11.11	174.98	88.16%	64.49%
Test group ^*^	0.657	12.13	171.66	81.55%	64.06%
Validation set	0.621	10.67	221.50	78.98%	66.48%

Abbreviations: MAE, mean absolute error; MSE, mean square error. Ensemble: LightGBM: GBRT: CatBoost=5: 3: 2; # Model including 20 variables after feature selection * Model including all of 24 variables ^a^ Absolute accuracy, the predicted trough concentration was within±20 mg/L of the observed trough concentration. ^b^ Relative accuracy, the predicted trough concentration was within±20% of the observed trough concentration.

The *R* ^*2*^ , MAE, MSE, absolute accuracy (±20 mg/L), and relative accuracy (±20%) of external validation were 0.621, 10.67, 221.50, 78.98% and 66.48%, respectively ( Table 3 ). The validation results shown in **Fig. S2** indicated quite a good generalization ability of the ensemble model, as shown in the comparison of predicted and observed values.

### Interpretation of the ensemble model

**Fig. 3:**
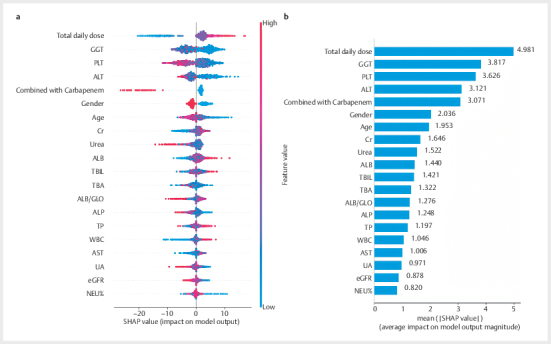
The ensemble model’s interpretation by SHAP. Abbreviations: SHAP: Shapley Additive exPlanations, VPA: valproic acid, GGT, γ-glutamyltransferase; PLT, platelet; ALT, alanine aminotransferase; AST, aspartate aminotransferase; ALP, alkaline phosphatase; TBIL, total bilirubin; TBA, total bile acid; TP, total protein; ALB, albumin; ALB/GLO, albumin/globulin; UA, uric acid; Cr, creatinine; eGFR, estimated glomerular filtration rate; WBC, white blood cell count; NEU%, the percentage of neutrophils. SHAP Summary Plot ( Fig. 3a ): The SHAP summary plot showcases the top 20 variables that significantly influence the prediction of VPA (Valproic acid) plasma concentration in the ensemble model. The x-axis represents the SHAP values, which are unified indices that quantify the impact of each variable on the model’s output. A higher SHAP value indicates a stronger influence on the predicted VPA concentration. Each row in the plot corresponds to a variable, with colored dots representing individual patient data points. Red dots signify higher values of the variable, while blue dots represent lower values. The color gradient helps interpret the combined effect of variable value and its SHAP value on the model’s prediction. For instance, variables with predominantly red dots and high SHAP values significantly increase the predicted VPA concentration. Importance Ranking of Variables ( Fig. 3b ): The bar chart in Fig. 3b ranks the top 20 variables based on the mean absolute SHAP values, providing a clear view of their relative importance to the model. The total daily dose of VPA administration is ranked first with a SHAP value of 4.981, indicating it has the most substantial impact on predicting VPA plasma concentration. This ranking helps identify the key predictors and their order of influence, crucial for understanding the model’s predictive logic.

Based on the selected variables, the SHAP data showed the correlation between the top 20 variables and VPA concentrations in the ensemble model ( Fig. 3a ). The eigenvalues ranked the importance of the 20 relevant variables for the prediction model, e. g., the total daily dose of VPA administration was ranked first, indicating that it had the largest impact on the prediction of VPA plasma concentration. The color of the dots indicated the eigenvalue of each variable, with increasing intensity of red color indicating higher eigenvalues, and increased intensity of blue color indicating lower eigenvalues. Each eigenvalue of a variable corresponded to a SHAP value of 1 on the x-axis. For instance, the SHAP values for each variable were summed to obtain the predicted VPA concentration. To identify the features with the greatest impact on the ensemble model, the average of the absolute SHAP values of the top 20 relevant variables with the highest eigenvalues was calculated. As shown, the top 20 variables were in descending order, and the SHAP value of the total daily dose of VPA administration had the highest score of 4.981, followed by those of GGT and PLT, at 3.817 and 3.626, respectively.

**Fig. 4:**
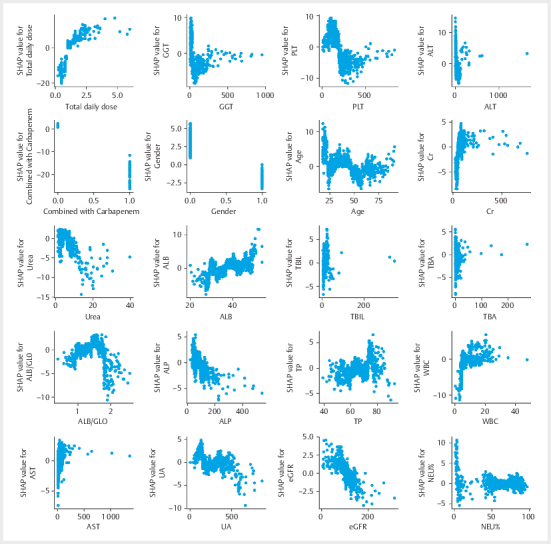
SHAP dependence plot of the ensemble model. Abbreviations: SHAP: Shapley Additive exPlanations, Combined with Carbapenem: 0 for no, 1 for yes. Gender, 0 for female, 1 for male. GGT, γ-glutamyltransferase; PLT, platelet; ALT, alanine aminotransferase; AST, aspartate aminotransferase; ALP, alkaline phosphatase; TBIL, total bilirubin; TBA, total bile acid; TP, total protein; ALB, albumin; ALB/GLO, albumin/globulin; UA, uric acid; Cr, creatinine; eGFR, estimated glomerular filtration rate; WBC, white blood cell count; NEU%, the percentage of neutrophils. The SHAP dependence plot showed how the relevant variable affected the output of the ensemble prediction model. SHAP values for specific relevant variables exceed 0, representing an increased teicoplanin plasma concentration.

Fig. 4 shows the SHAP dependence plot for the top 20 ranked variables of interest. ​The SHAP dependence plot of the variables showed that higher total daily dose, Cr, ALB, as well as lower PLT, urea, and eGFR were related to higher VPA plasma concentration. In addition, consistent with the literature 18 19 , female patients tend to have higher VPA plasma concentrations than men. The simultaneous use of carbapenems during the administration of VPA, however, resulted in a significant decrease in plasma concentration of VPA (41.54 mg/L±18.45 mg/L vs. 71.23 mg/L±20.70 mg/L, P<0.001).

### Software application for prediction

**Fig. 5:**
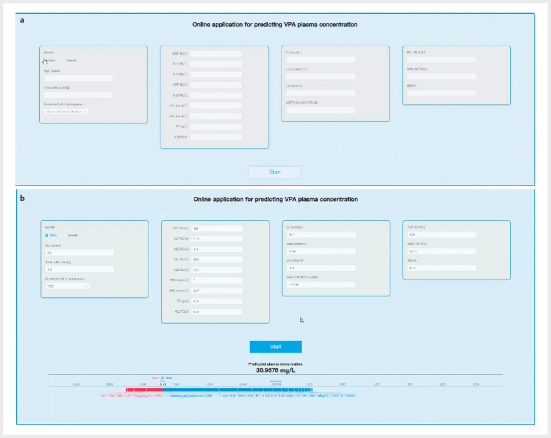
The interface of software application for calculating real-time prediction of VPA concentration. Abbreviations: VPA: valproic acid, GGT, γ-glutamyltransferase; PLT, platelet; ALT, alanine aminotransferase; AST, aspartate aminotransferase; ALP, alkaline phosphatase; TBIL, total bilirubin; TBA, total bile acid; TP, total protein; ALB, albumin; ALB/GLO, albumin/globulin; UA, uric acid; Cr, creatinine; eGFR, estimated glomerular filtration rate; WBC, white blood cell count; NEU%, the percentage of neutrophils. A: Original Interface: Fig. 5a displays the initial interface of the software application, where users can input patient biomarker data, including Total daily dose, gender, GGT, PLT, ALT, etc. B: Predicted Interface: Fig. 5b shows the interface post-prediction, exemplified by the data of the first patient. After inputting specific values (e. g., gender, co-administration of Carbapenem, GGT value), the system provides the predicted VPA plasma concentration based on the ensemble model. Additionally, detailed SHAP interpretations are presented, highlighting the influential factors affecting the prediction.

As shown in Fig. 5 , a real-time calculator for VPA plasma concentration prediction based on the model was devolved. This user-friendly tool allows clinicians to input patient data, and the prediction is generated automatically. Besides, missing values are accepted, as the model can automatically complete the required information. Meanwhile, the corresponding force plot was also demonstrated, which enables clinicians to gain a better understanding of how each feature influences the model’s decision, thereby enhancing user comprehension and trust. Currently, the software has been deployed on the intranet of our hospital, allowing clinicians to access it.

## Discussion

With the rapid development of ML, it is now widely used in biomedical fields such as clinical diagnosis, precision therapy, and health monitoring 20 . We constructed an optimal prediction model for the VPA concentration and interpreted the prediction model using the SHAP method. Based on the model, we also developed VPA concentration prediction software that allows for timely prediction of VPA concentration by entering relevant clinical parameters, enhancing convenience and efficiency. Furthermore, after the software is launched, it will incorporate incremental learning from real-world clinical data and continuously optimize the model through clinical practice validation, ensuring safe and rational medication for patients with epilepsy.

Our results show that the total daily dose of VPA ranks first in the SHAP value. Some studies suggest that the total daily dose can be increased by 5 to 10 mg/kg/d on a weekly basis, up to a maximum of 60 mg/kg/d, within a target therapeutic range of 50 to 100 mg/L 21 22 . This implies that within a certain range, an appropriate increase in the total daily dose of VPA administered is beneficial for clinical efficacy. The presence of two distinct populations in Fig. 3a can be explained by the method of calculating SHAP values. SHAP values measure the marginal contribution of each feature to the model’s prediction, considering complex interactions among features. In the lower dosage range, the impact of VPA on pharmacokinetic parameters is minimal, resulting in lower and negative SHAP values, forming the first population. In the higher dosage range, the impact of VPA significantly increases, resulting in higher and positive SHAP values, forming the second population. This phenomenon reflects different pharmacokinetic behaviors of VPA across various dosage ranges, consistent with the pharmacokinetic principles proposed by Hiemke et al. 23 . VPA is mainly metabolized in the liver; therefore, hepatic insufficiency slows its metabolism 24 . Notably, the concomitant use of VPA with drugs that have a high risk of hepatotoxicity, such as acetaminophen and nonsteroidal anti-inflammatory drugs (NSAIDs), results in impaired drug metabolism. Co-administration of VPA with hepatic enzyme inducers decreases VPA concentrations, whereas co-administration with hepatic enzyme inhibitors results in increased VPA concentrations. Therefore, pharmacists should intensify VPA monitoring efforts to ensure its efficacy and safety, particularly in patients receiving multiple medications simultaneously.

Consistent with our findings, several studies have reported that the combination of carbapenems and VPA reduces the latter concentration 25 26 and increases the risk of epilepsy. In our study, a total of 93 patients were treated with VPA in combination with carbapenems and failed to achieve VPA efficacy. The potential mechanisms by which carbapenems may influence plasma concentrations of VPA include inhibiting valproate uptake in the enterocytes of the small intestinal villi 24 26 and blocking carriers of multidrug resistance-associated proteins (MRPs), thereby affecting VPA distribution 24 27 . Additionally, carbapenems can impact the metabolism and excretion of VPA 28 29 . Given their ability to rapidly and significantly reduce VPA levels, concurrent use of carbapenems with VPA should be avoided.

In our study, 65 patients received combined aspirin (ASA). ASA irreversibly inhibits platelet adhesion and aggregation by acetylating the essential functional serine on platelet cyclooxygenase and affects approximately 40% of VPA metabolism as an inhibitor of β-oxidation 30 . Decreased PLT counts, associated with reduced clearance of voriconazole, may have similar effects on VPA 31 32 . Thus, clinical administration of VPA should include monitoring of PLT changes and avoiding the concurrent use of ASA. Additionally, protein binding, a dynamic process varying among patients, affects VPA concentration. The plasma protein binding rate of VPA is 85%–95% 4 8 , with ALB, ALB/GLO, and TP closely related to VPA levels. Reduced albumin increases the unbound fraction of VPA, potentially causing adverse reactions 33 . Therefore, in patients with hypoalbuminemia, albumin supplementation should be prioritized for effective drug therapy and to maintain normal physiological functions.

VPA is primarily excreted by the kidneys, with a small amount excreted in the feces. Chronic renal hypofunction with low plasma protein levels may lead to elevated free VPA concentrations and possibly accelerated metabolic excretion 34 35 . The metabolism of VPA, primarily in the liver, involves multiple cytochrome P450 enzymes, chiefly CYP2A6, CYP2B6, CYP2C9, and CYP2C19 23 . This metabolic interplay notably influences the pharmacokinetics of VPA. Although our retrospective study did not analyze genetic data directly, accounting for this variability in clinical settings is critical to optimize dosing strategies and ensure the attainment of targeted therapeutic levels. Studies have shown that parameters related to renal function, including Cr, urea, UA, and eGFR, are all correlated with the concentration of VPA, which is consistent with the findings of our study. However, renal dysfunction leads to a slower excretion of VPA, a longer elimination half-life, and increased plasma concentrations. The co-administration of VPA with drugs with a high risk of nephrotoxicity can also increase the metabolic burden on renal function and affect VPA concentration.

Our study found that VPA concentrations are higher in female patients than male patients, as reported in other studies 18 19 35 . The correlation between VPA metabolism, concentration, and gender remains controversial. Some studies suggest that gender differences in VPA pharmacokinetics may be primarily due to variations in UDP-glucuronosyltransferase activity, which metabolizes approximately 40% of VPA 19 35 . Additionally, gender significantly affects the disposition parameters through different hepatobiliary transfers of VPA, resulting in a higher reabsorbed fraction in women 36 . However, few large-scale, real-world studies have investigated gender differences in VPA concentration. Thus, more robust evidence is needed to elucidate gender-related clinical concentrations of VPA.

Our research demonstrates that WBC and NEU% are the factors influencing VPA plasma concentration. WBC accounts for more than half of the total number of leukocytes, and the elevated levels of WBC and NEU% in the organism are mostly indicative of inflammation and tissue damage due to bacterial infection 37 . Significant changes affecting drug concentrations are linked to the body’s water dynamics. Excess water shifts into the extracellular space, thereby increasing the apparent volume of drug distribution and diluting VPA concentration. Additionally, increased renal filtration driven by elevated cardiac output can lead to a higher elimination rate of drugs 38 . As a hydrophilic drug, VPA may dilute due to increased extracellular fluid from infection and enhanced renal filtration.

Several limitations of our study should be noted. First, this is a retrospective study conducted in a single center; therefore, additional studies are needed to determine the applicability of this model to different populations and regions. Second, due to the use of static datasets, our model lacked the ability to update online. Moreover, the incremental learning capacity of the model is currently limited. While we have implemented feature importance analysis and parameter adjustment to enhance predictive accuracy, the model’s ability to adapt to new data and refine predictions incrementally remains an area for future improvement. We are currently working on integrating the calculator into the TDM platform to achieve self-learning and iterative optimization, which will allow for incremental learning using real-time collection of new samples in future studies. Additionally, the predictive accuracy and feature importance analysis of this model are based on a specific dataset, which may limit generalizability to different contexts or populations. Notably, while achieving a plasma concentration of 50 to 100 mg/L is typically within the target therapeutic range, it does not guarantee efficacy for all patients due to pharmacodynamic variability and individual differences, such as genetic factors, hepatic and renal function, drug interactions, and age. This underscores the need for careful clinical assessment beyond merely achieving target plasma concentrations.

Despite limitations, our study offers an efficient, cost-effective VPA concentration prediction using SHAP, enhancing model transparency and understanding the impacts of clinical factors. Introducing VPApredictor1.0, a user-friendly web app with SHAP-integrated insights, provides real-time, personalized dose adjustments, distinguishing from standard pharmacokinetic models. Moreover, by leveraging multi-algorithm mining techniques and SHAP-based interpretability, our approach can be generalized to predict concentrations of other antiepileptic drugs and optimize chronic disease management. The adaptive capabilities of our prediction tool hold promise for personalized medicine in chronic diseases, where accurate dosage and continuous drug monitoring are critical for effective treatment outcomes.

## Data availability statement

Data are available upon request from the corresponding author.

## Author contributions

All named authors meet the International Committee of Medical Journal Editors (ICMJE) criteria for authorship for this article. J.X., P.M., and Y.C. designed the research; X.P., C.L., and R.L. performed the analyses; L.X., H.W., and X.Z. collected and interpreted data; J.X., P.M., and L.X. wrote the paper; J.X., P.M., X.P., C.L., R.L., L.X., H.W., X.Z., L.X., and Y.C. critically revised the paper; L.X. and Y.C. final approved of the version to be published. All authors contributed to the article and approved the submitted version.

## Funding

Science and Health Joint Medical Research Project of Chongqing (2023QNXM031)
