# Supplementary material for: Ensemble Machine Learning Model for Real-Time Valproic Acid Prediction in Epilepsy Treatment
Source: Pharmacopsychiatry. 2025 Jun 2;59(3):145–56. doi: 10.1055/a-2593-3125 (PMC13288425; doi:10.1055/a-2593-3125)
Supplement: Supplementary file 1 — Supplementary Material [file 10-1055-a-2593-3125-php-2024-07-1296.pdf]

Supplementary Data

Table S1. The proportional combinations of the three algorithms and the corresponding R^2 of each combination.

Algorithms Ratio	R^2	Algorithms Ratio	R^2	Algorithms Ratio	R^2	Algorithms Ratio	R^2	Algorithms Ratio	R^2	Algorithms Ratio	R^2
0.0: 0.0: 1.0	0.5202	0.1: 0.0: 0.9	0.5297	0.2: 0.1: 0.7	0.5427	0.3: 0.3: 0.4	0.5532	0.4: 0.6: 0.0	0.5509	0.6: 0.4: 0.0	0.5559
0.0: 0.1: 0.9	0.5262	0.1: 0.1: 0.8	0.5353	0.2: 0.2: 0.6	0.5463	0.3: 0.4: 0.3	0.5533	0.5: 0.0: 0.5	0.5505	0.7: 0.0: 0.3	0.5504
0.0: 0.2: 0.8	0.5307	0.1: 0.2: 0.7	0.5394	0.2: 0.3: 0.5	0.5484	0.3: 0.5: 0.2	0.5520	0.5: 0.1: 0.4	0.5543	0.7: 0.1: 0.2	0.5534
0.0: 0.3: 0.7	0.5337	0.1: 0.3: 0.6	0.5419	0.2: 0.4: 0.4	0.5490	0.3: 0.6: 0.1	0.5492	0.5: 0.2: 0.3	0.5566	0.7: 0.2: 0.1	0.5548
0.0: 0.4: 0.6	0.5352	0.1: 0.4: 0.5	0.5430	0.2: 0.5: 0.3	0.5481	0.3: 0.7: 0.0	0.5448	**0.5: 0.3: 0.2**	**0.5574**	0.7: 0.3: 0.0	0.5548
0.0: 0.5: 0.5	0.5352	0.1: 0.5: 0.4	0.5425	0.2: 0.6: 0.2	0.5457	0.4: 0.0: 0.6	0.5479	0.5: 0.4: 0.1	0.5568	0.8: 0.0: 0.2	0.5477
0.0: 0.6: 0.4	0.5336	0.1: 0.6: 0.3	0.5406	0.2: 0.7: 0.1	0.5418	0.4: 0.1: 0.5	0.5522	0.5: 0.5: 0.0	0.5546	0.8: 0.1: 0.1	0.5503
0.0: 0.7: 0.3	0.5306	0.1: 0.7: 0.2	0.5371	0.2: 0.8: 0.0	0.5364	0.4: 0.2: 0.4	0.5549	0.6: 0.0: 0.4	0.5513	0.8: 0.2: 0.0	0.5513
0.0: 0.8: 0.2	0.5260	0.1: 0.8: 0.1	0.5321	0.3: 0.0: 0.7	0.5436	0.4: 0.3: 0.3	0.5562	0.6: 0.1: 0.3	0.5547	0.9: 0.0: 0.1	0.5434
0.0: 0.9: 0.1	0.5200	0.1: 0.9: 0.0	0.5256	0.3: 0.1: 0.6	0.5483	0.4: 0.4: 0.2	0.5559	0.6: 0.2: 0.2	0.5566	0.9: 0.1: 0.0	0.5455
0.0: 1.0: 0.0	0.5124	0.2: 0.0: 0.8	0.5375	0.3: 0.2: 0.5	0.5515	0.4: 0.5: 0.1	0.5542	0.6: 0.3: 0.1	0.5570	1.0: 0.0: 0.0	0.5372

Computer grid search was employed on the weight proportion (accurate to one decimal place) of the three algorithms, and the R^2 of each model were calculated. The final ensemble model was determined as the model with the highest R^2. the composition of LightGBM, GBRT and CatBoost (5:3:2) with the highest R^2 was determined as the final ensemble model.

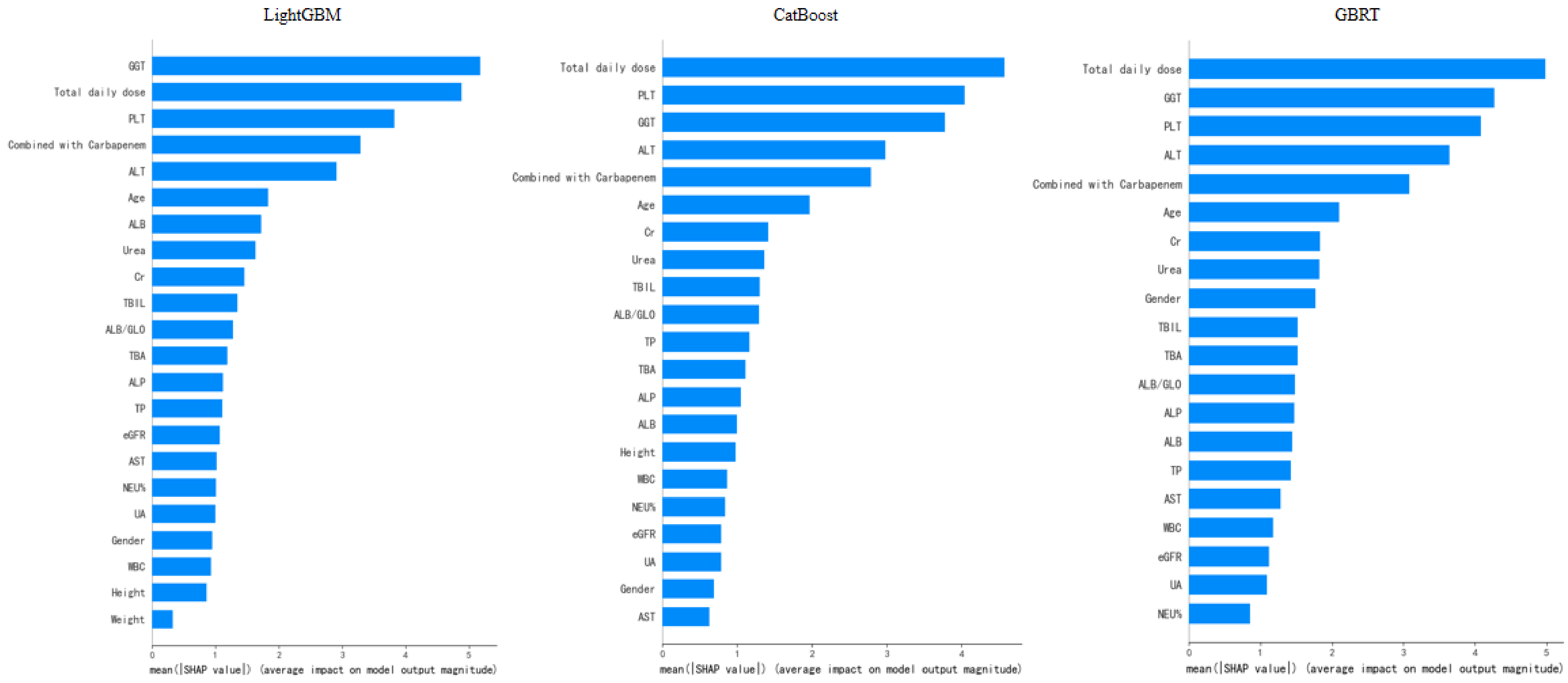

Figure S1. Feature sets mined by three algorithms.

Abbreviations: LightGBM, light gradient boosting machine; CatBoost, categorical boosting; GBRT, gradient boosted regression trees. Each algorithm selects the feature that will reach the highest R^2 from all the features as the feature mined by the algorithm. GGT, γ-glutamyltransferase; PLT, platelet; ALT, alanine aminotransferase; AST, aspartate aminotransferase; ALP, alkaline phosphatase; TBIL, total bilirubin; TBA, total bile acid; TP, total protein; ALB, albumin; ALB/GLO, albumin/globulin; UA, uric acid; Cr, creatinine; eGFR, estimated glomerular filtration rate; WBC, white blood cell count; NEU%, the percentage of neutrophils.

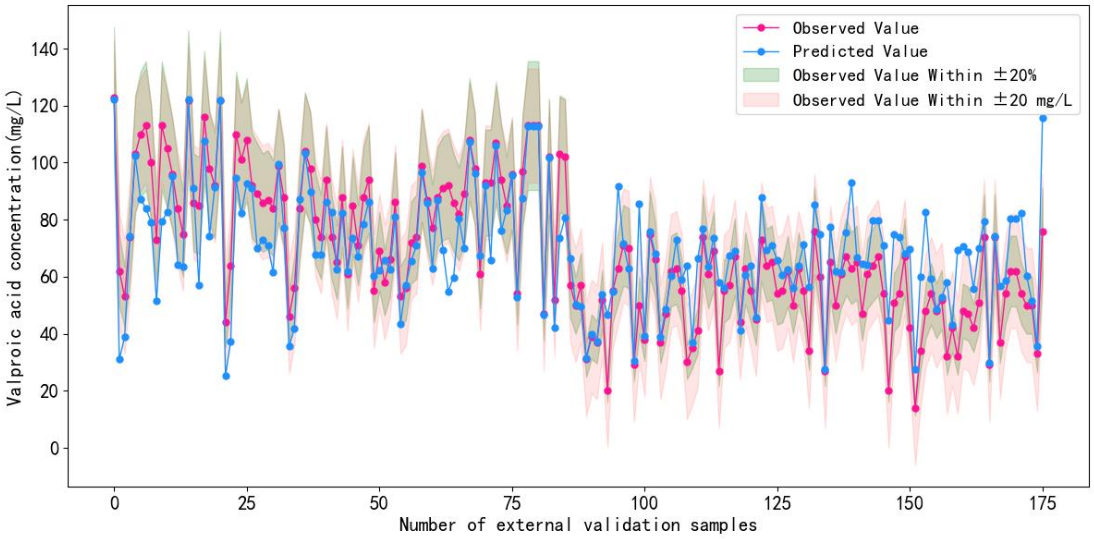

Figure S2. Comparison of predicted and observed value in validation set.

The red dots indicated the observed values, and blue dots indicated the predicted values. The pink shade represented within ±20% of the observed values, and the purple shade represented within ±20mg/L of the observed values.
